# Synthesis, Property Characterization and Photocatalytic Activity of the Polyaniline/BiYTi_2_O_7_ Polymer Composite

**DOI:** 10.3390/polym9030069

**Published:** 2017-02-23

**Authors:** Jingfei Luan, Yue Shen, Shu Wang, Ningbin Guo

**Affiliations:** State Key Laboratory of Pollution Control and Resource Reuse, School of the Environment, Nanjing University, Nanjing 210093, China; yueshen_sally@outlook.com (Y.S.); wshgod@126.com (S.W.); MG1325044@smail.nju.edu.cn (N.G.)

**Keywords:** polyaniline/BiYTi_2_O_7_, photocatalytic activity, Azocarmine G, visible light irradiation, H_2_O_2_

## Abstract

A new polyaniline/BiYTi_2_O_7_ polymer composite was synthesized by chemical oxidation in-situ polymerization method for the first time. The effect of polyaniline doping on structural and catalytic properties of BiYTi_2_O_7_ was reported. The structural properties of novel polyaniline/BiYTi_2_O_7_ have been characterized by X-ray diffraction, scanning electron microscopy, X-ray photoelectron spectroscopy and UV-Vis DRS. The results showed that BiYTi_2_O_7_ crystallized well with the pyrochlore-type structure, stable cubic crystal system by space group Fd3m. The lattice parameter or band gap energy of BiYTi_2_O_7_ was found to be *a* = 10.2132 Å or 2.349 eV, respectively. The novel polyaniline/BiYTi_2_O_7_ polymer composite possessed higher catalytic activity compared with BiYTi_2_O_7_ or nitrogen doped TiO_2_ for photocatalytic degradation of Azocarmine G under visible light irradiation. Additionally, the Azocarmine G removal efficiency was boosted from 3.0% for undoped BiYTi_2_O_7_ to 78.0% for the 10% polyaniline-modified BiYTi_2_O_7_, after only 60 min of reaction. After visible light irradiation for 330 min with polyaniline/BiYTi_2_O_7_ polymer composite as photocatalyst, complete removal and mineralization of Azocarmine G was observed. The photocatalytic degradation of Azocarmine G followed first-order reaction kinetics. Ultimately, the promoter action of H_2_O_2_ for photocatalytic degradation of AG with BiYTi_2_O_7_ as catalyst in the wastewater was discovered.

## 1. Introduction

The pollution of water resources by large quantities of azo-dyes effluents, generated from diverse industries such as chemical, textile or printing, due to their toxicity, high chemical oxygen demand content, and biological degradation, has a dramatically negative environmental impact, affecting the quality of drinking water. In the last decade, photocatalytic degradation processes had been widely applied as techniques for the destruction of organic pollutants in wastewater and effluents [1,2,3,4,5,6,7,8,9,10,11,12,13,14,15,16,17,18,19,20]. The photocatalytic degradation process has several advantages over other competing processes such as complete mineralization, no waste disposal problem, low cost, and only needing mild temperature and pressure [8].

**Figure 1:**
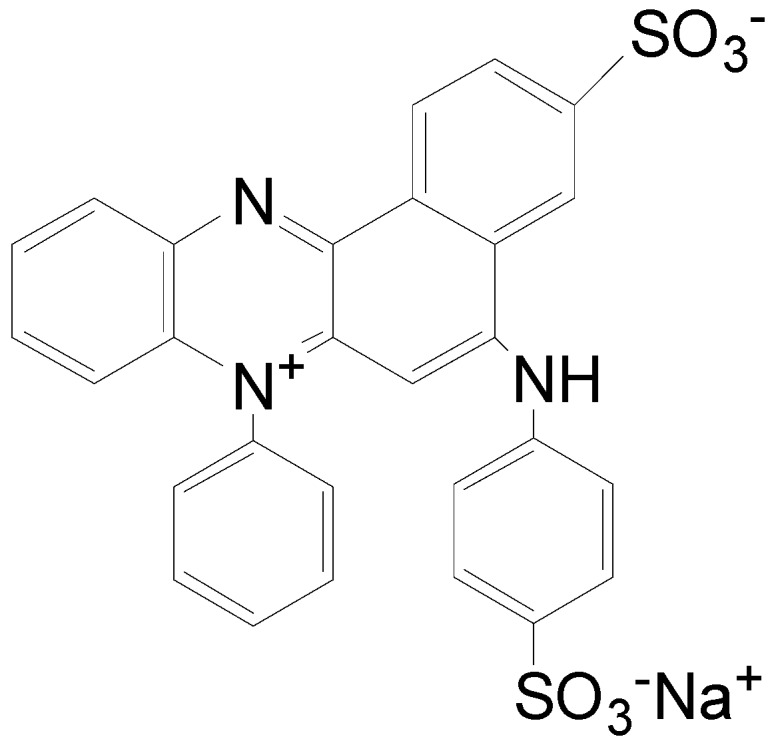
The molecular structure of Azocarmine G.

Azocarmine G (AG) (Figure 1) as a common biological stain is often used to determine protein [21], DNA [22] and light neurons [23]. AG, a common kind of azo dye, is more difficult to handle. In exceptional circumstances, azo dyes can decompose to produce over 20 kinds of carcinogenic aromatic amines, which can cause lesions and induce cancer after activation to change the DNA structure of the human body. Since the Sudan Red incident occurred, the use of biological stain and the wastewater treatment has been more cautious to the researchers. AG, however, is harder to be biodegraded and photodegraded directly. Many methods on the photodegradation of AG are reported. Unfortunately, most of these experiments were carried out under ultraviolet light irradiation. Nowadays, there are only a few reports for AG dye degradation under visible light irradiation, finding that the removal ratio of AG is very low.

It is known that ultraviolet light only occupies 4% of the solar energy spectrum. For this reason, there is great interest in developing new visible light-responsive photocatalysts which are capable of utilizing the more ample visible light spectrum, which occupies about 43% of the solar energy range. Therefore, it is an urgent need to develop novel visible light-responsive photocatalysts. According to the past articles [24,25,26,27,28,29], many semiconductors can be used as photocatalysts under ultraviolet light irradiation, such as TiO_2_ and ZnO.

In view of the efficient utilization of solar energy, numerous attempts have been made in recent years to develop different visible light-active photocatalysts [30,31,32,33,34,35,36,37,38,39,40,41,42,43,44,45,46,47]. Traditionally, for improving the photocatalytic efficiency of photocatalysts, the bandgap of photocatalysts or loaded cocatalysts plays an important role in photocatalyst system. Therefore, efficient catalysts that can generate electron–hole pairs under visible light irradiation should be developed [48,49,50,51,52,53,54,55,56,57,58,59,60,61,62,63,64]. Among them, A_2_B_2_O_7_ compounds with narrow band gaps have been proven to be good candidates for photocatalytic degradation of organic pollutants under visible light irradiation. In our previous work [65], we found that In_2_YbSbO_7_ and Gd_2_YbSbO_7_ which owned the pyrochlore-type structure as a photocatalyst under visible light irradiation seemed to be potential for improvement of photocatalytic activity by modifying its structure.

A change and improvement of the electron transport and photophysical properties could be found in the novel BiYTi_2_O_7_ compound which might display advanced photocatalytic properties. BiYTi_2_O_7_ had never been produced and the data about its structural such as space group and lattice constants had not been previously reported. In addition, the photocatalytic properties of BiYTi_2_O_7_ had not been investigated by other investigators. The molecular composition of BiYTi_2_O_7_ was very similar to that of other A_2_B_2_O_7_ compounds. BiYTi_2_O_7_ also seemed to have potential for improvement of photocatalytic activity by modifying its structure, because it had been proven that a slight modification of a semiconductor structure would cause a remarkable change in photocatalytic properties.

Polyaniline (PANI) has a high application rate in view of its electrical and optical properties, ease of derivatization, solubility in several solvents, processability inside fibers and films, and stability [66,67,68]. In addition, PANI with delocalized π-conjugated structures is beneficial to rapid charge separation, thus the separation efficiency of photogenerated electron (e^−^)–hole (h^+^) pairs can be significantly improved [69]. Shang et al. [70] reported that on the basis of the small grain size, the intrinsic property of PANI, and the synergic effect between PANI and BiVO_4_, a rapid electron–hole separation and slow recombination was realized. Hidalgo et al. [71] prepared PANI–TiO_2_ nanocomposites by a novel and green sol-gel spin coating method. The results showed that the photocurrent in PANI/TiO_2_ electrodes dramatically increased under simulated sunlight irradiation, reaching maximum photocurrent densities around 2 and 1.6 fold higher than the pristine TiO_2_ NPs.

Moreover, due to the excellent environmental stability of polyaniline, the polyaniline–hybridized BiYTi_2_O_7_ sample should possess more advanced photocatalytic properties. Thus, the resemblance suggested that BiYTi_2_O_7_ and the polyaniline–hybridized BiYTi_2_O_7_ may own photocatalytic properties under visible light irradiation, like other members in the A_2_B_2_O_7_ family.

For the purpose of further designing novel high–efficiency and stable photocatalysts, in this study, polyaniline with ultra-low loading amount (1%, 5% and 10%) was loaded onto BiYTi_2_O_7_ to synthesize polyaniline/BiYTi_2_O_7_ polymer composites by the chemical oxidation polymerization method for the first time. The structural and photocatalytic properties of the polyaniline–hybridized BiYTi_2_O_7_ were studied in detail. The photocatalytic property was evaluated by the degradation of AG under visible light irradiation. The 10% polyaniline–hybridized BiYTi_2_O_7_ exhibited excellent photocatalytic activity. In order to elucidate the structure–photocatalytic activity relationship in the polyaniline–hybridized BiYTi_2_O_7_, a comparison among the photocatalytic properties of the polyaniline–hybridized BiYTi_2_O_7_, BiYTi_2_O_7_ and nitrogen doped TiO_2_ (N-doped TiO_2_) was carried out. The reaction mechanism of the improved photocatalytic performance of polyaniline/BiYTi_2_O_7_ polymer composites was also investigated.

## 2. Materials and Methods

### 2.1. Preparation Method of BiYTi_2_O_7_

The novel photocatalyst had been synthesized by solid-state reaction method. Bi_2_O_3_, Y_2_O_3_, and TiO_2_ with purity of 99.99% (Sinopharm Group Chemical Reagent Co., Ltd., Shanghai, China) were used as starting materials without further purification. Owing to the volatility of Bi_2_O_3_, the molar ratio of atoms in BiYTi_2_O_7_ (*n*(Bi):*n*(Y):*n*(Ti):*n*(O)) does not close to the stoichiometric ratio of 1:1:2:7 after the experiment, therefore, we finally decided to add 130% quantities of Bi_2_O_3_ after 5 experiments to gain pure BiYTi_2_O_7_ catalyst. All powders (*n*(Bi_2_O_3_):*n*(Y_2_O_3_):*n*(TiO_2_) = 1.3:1:4) were dried at 200 °C for 4 h before synthesis. In order to synthesize BiYTi_2_O_7_, the precursors were stoichiometrically mixed, then pressed into small columns and put into an alumina crucible (Shenyang Crucible Co., Ltd., Shenyang, China). After the raw materials calcining at 400 °C for 2 h, then at 750 °C for 10 h, we took the small columns out of the electric furnace, ground the mixed materials and subsequently put them into the electric furnace (KSL 1700X, Hefei Kejing Materials Technology Co., Ltd., Hefei, China). The mixed materials were calcined at 1050 °C for 30 h with an intermediate regrinding process in an electric furnace. Finally, pure BiYTi_2_O_7_ catalyst, which presented the color of light yellow, was obtained after total grinding. One hundred-gram samples were synthesized at a time.

### 2.2. Preparation of Polyaniline–Hybridized BiYTi_2_O_7_

An amount of distilled aniline (0.2, 1 or 2 g) was added to 150 mL of 1M HCl, and subsequently stirred for 30 min to ensure that the aniline was totally dissolved. Subsequently, a certain percentage of BiYTi_2_O_7_ (19.8, 19 or 18 g, respectively) was added into above solution, sonicated for 30 min to obtain a dispersed solution, and then stirred for 1 h. Thirdly, 0.5 g·mL^−1^ ammonium thiosulfate (HCl) was added into the solution slowly; subsequently, the mixture was stirred for 24 h with the final color of deep green. Finally, the suspension was filtered, and the precipitate was washed with alcohol and water for many times and dried at 60 °C to obtain polyaniline (PANI)–hybridized-BiYTi_2_O_7_. In this article, we named the photocatalysts which were m(PANI):m(BiYTi_2_O_7_) = 1:100, 5:100, and 10:100 as BiYTi_2_O_7_-1%PANI, BiYTi_2_O_7_-5%PANI, and BiYTi_2_O_7_-10%PANI, respectively. The batch size among three synthesized samples was 20 g at a time.

### 2.3. Characterization of BiYTi_2_O_7_ and PANI–BiYTi_2_O_7_

The crystal structures of BiYTi_2_O_7_ and PANI–BiYTi_2_O_7_, were analyzed by the powder X-ray diffraction method (D/MAX-RB, Rigaku Corporation, Tokyo, Japan) with Cu*K*α radiation (λ = 1.54056). The data were collected at 295 K with a step-scan procedure in the range of 2θ = 10°–100°. The step interval was 0.02° and the time per step was 1.2 s. The chemical composition of the compound was determined by Scanning Electron Microscope-X-ray Energy Dispersion Spectrum (SEM-EDS, LEO 1530VP, LEO Corporation, Dresden, Germany). The Bi^3+^ content, Y^3+^ content, Ti^4^^+^ content, and O^2−^ content of BiYTi_2_O_7_ were determined by X-ray photoelectron spectroscopy (XPS, ESCALABMK-2, VG Scientific Ltd., London, UK). The chemical composition within the depth profile of BiYTi_2_O_7_ was examined by the argon ion denudation method when X-ray photoelectron spectroscopy was used. The optical absorption of BiYTi_2_O_7_ and PANI–BiYTi_2_O_7_ was analyzed with an UV-Visible spectrophotometer (UV-2450, Shimadzu Corporation, Kyoto, Japan). The particle sizes of the photocatalysts were determined by Malvern’s Mastersize-2000 particle size analyzer (Malvern Instruments Ltd., Malvern, UK).

### 2.4. Photocatalytic Characterizations of BiYTi_2_O_7_ and PANI–BiYTi_2_O_7_

The photocatalytic degradation experiments of AG were carried out with photocatalyst powders which were suspended in 50 mL 20 mg·L^−1^ solution in 12 identical Pyrex glass cells (Nanjing Xujiang Industry, Nanjing, China). Before light irradiation, the suspensions were magnetically stirred in the darkness for 30 min to ensure establishment of an adsorption-desorption equilibrium among photocatalyst, the dye and atmospheric oxygen. The photocatalytic reaction system consisted of a 500 W Xe arc lamp (Nanjing Xujiang Industry, Nanjing, China), a magnetic stirrer and a cut-off filter (λ > 420 nm, Nanjing Xujiang Industry, Nanjing, China). The Xe arc lamp was surrounded by a quartz jacket and was positioned in a photoreactor quartz vessel. Room temperature (25 °C) was maintained by an outer recycling water glass jacket. The solution was continuously stirred magnetically. After every 30 min, one of the Pyrex glass cells was sampled. Without pH adjustment, the initial pH value was 6.0 for AG. Additionally, the concentration of AG was determined according to the absorption wavelength at 524 nm as measured by a UV-Vis spectrophotometer (UV-2550, Shimadzu Corporation, Kyoto, Japan), since the highest absorption wavelength of AG was 524 nm. The total organic carbon (TOC) concentration was determined with a TOC analyzer (TOC-5000, Shimadzu Corporation).

## 3. Results and Discussion

### 3.1. Characterization

#### 3.1.1. XRD Analysis

**Figure 2:**
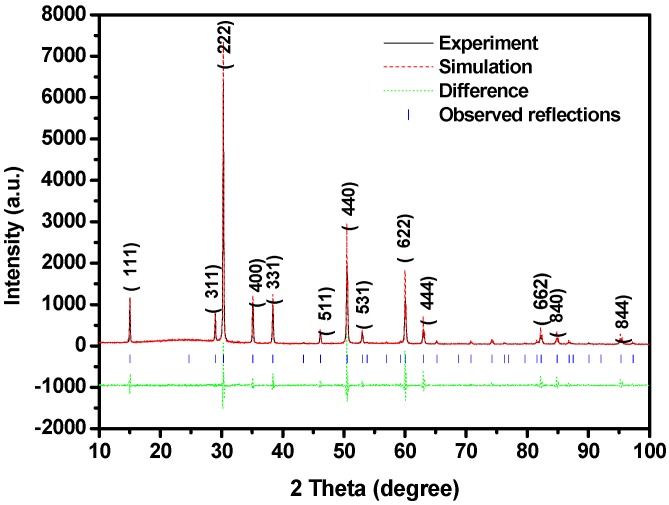
X-ray powder diffraction patterns and Rietveld refinements of BiYTi_2_O_7_ (black solid line represents experimental XRD data; red dotted line represents simulative XRD data; green dashed line represents a difference between experimental XRD data and simulative XRD data; and blue vertical line represents observed reflection positions).

The structure with full-profile refinements of the as-prepared product BiYTi_2_O_7_ was examined by X-ray diffraction technique and the results were shown in Figure 2. The collected data was obtained by the Materials Studio program, which was based on Rietveld analysis. The powder X-ray diffraction analysis revealed that the product could be melted when sintering temperature was over 1150 °C. In the light of Figure 2, it could be concluded that BiYTi_2_O_7_ was single phase and that the lattice parameter for this new photocatalyst BiYTi_2_O_7_ was 10.21323 Å. Simultaneously, the final refinement for BiYTi_2_O_7_ showed a good agreement between the observed and calculated intensities for the pyrochlore-type structure, a cubic crystal system and a space group Fd3m (O atoms are included in the model). In addition, all of the diffraction peaks for this photocatalyst could be indexed successfully, according to the lattice constant and above space group. Moreover, the atomic coordinates and structural parameters of BiYTi_2_O_7_ are recorded in Table 1.

As can be seen from the X-ray diffraction, it could be concluded that BiYTi_2_O_7_ crystallized into pyrochlore-type structure. The cubic system structure with space group Fd3m for Bi_2_Ti_2_O_7_ keeped unchanged after Bi^3+^ being substituted by Y^3+^. The results of full-profile structure refinements for BiYTi_2_O_7_ generated the unweighted *R* factors, *R*_P_ = 13.80% with space group Fd3m. Contrary to the work of Zou et al. [72], Bi_2_InNbO_7_ which was slightly modified had a larger *R* factor.

**Figure 3:**
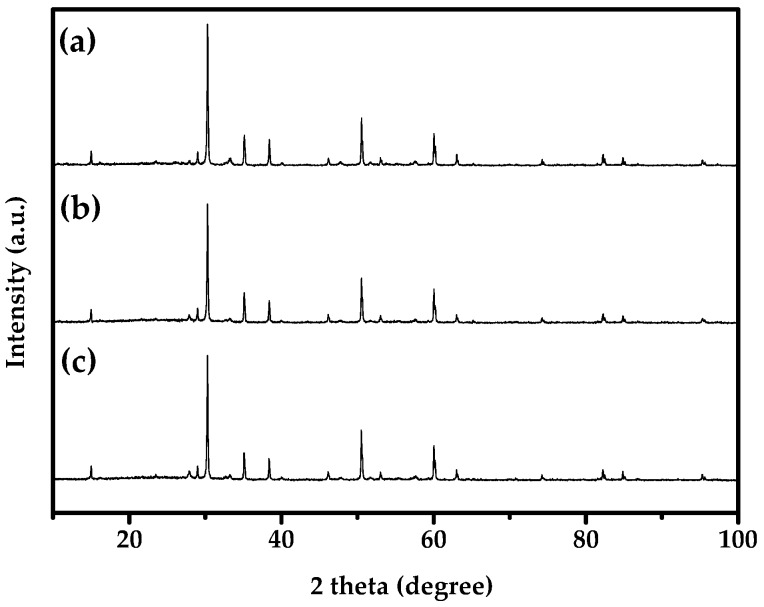
X-ray diffraction patterns of: (**a**) BiYTi_2_O_7_-1%PANI; (**b**) BiYTi_2_O_7_-5%PANI; and (**c**) BiYTi_2_O_7_-10%PANI.

Figure 3 presents the XRD pattern of different polyaniline based on BiYTi_2_O_7_. As shown in Figure 3, all the diffraction peaks of different polyaniline loaded on BiYTi_2_O_7_ were absolutely similar to BiYTi_2_O_7_ with pyrochlore-type structure. It could reveal that the structures of different polyaniline based on BiYTi_2_O_7_ were determined by the cubic structure of BiYTi_2_O_7_. The addition of polyaniline would not change the lattice structure of BiYTi_2_O_7_. In addition, the polyaniline parcel layer was very thin because we could not detect the diffraction peaks of polyaniline [73].

#### 3.1.2. SEM Analysis

**Figure 4:**
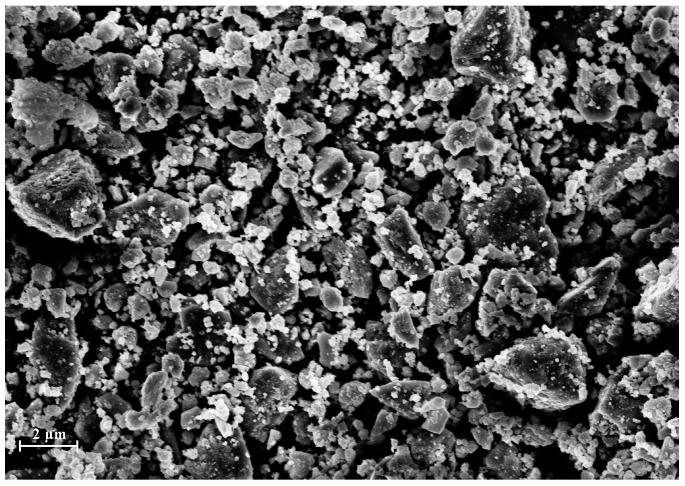
SEM spectra of BiYTi_2_O_7_.

**Figure 5:**
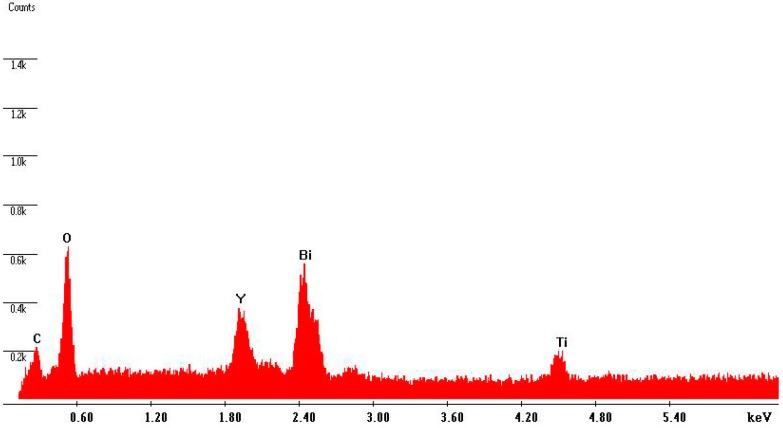
EDS spectra of BiYTi_2_O_7_.

The SEM image of BiYTi_2_O_7_ (Figure 4) shows that the BiYTi_2_O_7_ particle size is relatively uniform. The SEM-EDS spectrum of BiYTi_2_O_7_ (Figure 5) revealed that BiYTi_2_O_7_ was pure phase without any other impurities but bismuth, yttrium, titanium and oxygen (where the C peak was introduced by the necessary conductive paste in the energy spectrum analysis). Moreover, the molar ratio of its atoms (*n*(Bi):*n*(Y):*n*(Ti):*n*(O)) closed to the stoichiometric ratio of 1:1:2:7. It was impossible that the space groups we observed were generated from impurities. Therefore, it could conclude that the slight *R* factor of this photocatalyst was resulted from slightly modified structure model. Furthermore, the change of structures, including different bond-distance distributions, thermal displacement parameters and/or occupation factors for some of the atoms, were due to the defects or the disorder/order of a part of the atoms [74].

**Figure 6:**
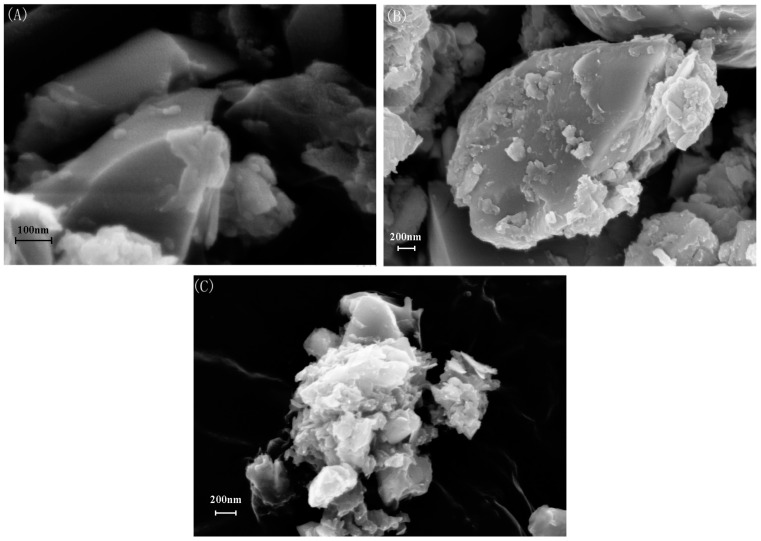
SEM images of: (**A**) BiYTi_2_O_7_-1%PANI; (**B**) BiYTi_2_O_7_-5%PANI; and (**C**) BiYTi_2_O_7_-10%PANI.

The SEM images of different polyaniline based on BiYTi_2_O_7_ (Figure 6) presented that, with the increase in the amount of polyaniline doped on the surface of BiYTi_2_O_7_ particle, the composite photocatalyst turned into coarser and coarser gradually. The layered material on the surface of BiYTi_2_O_7_ particle was polyaniline, adsorped on BiYTi_2_O_7_ particles by physical adsorption. Moreover, the SEM-EDS spectrum of different polyaniline based on BiYTi_2_O_7_ also revealed that the photocatalysts were pure phase without any other impurities but bismuth, yttrium, titanium, carbon, nitrogen and oxygen.

#### 3.1.3. XPS Analysis

**Figure 7:**
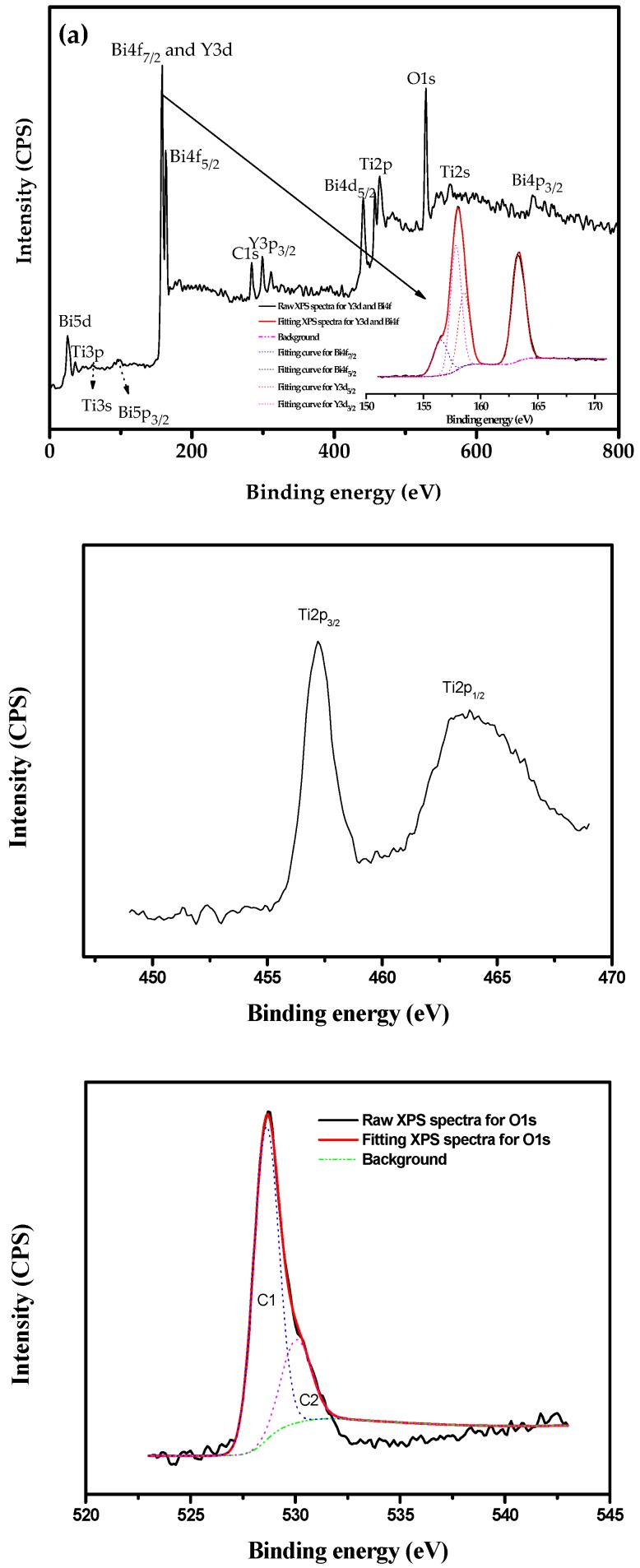
(**a**) Full XPS regions for BiYTi_2_O_7_ (Inset is the enlarged range of the Bi4f and Y3d peak); XPS spectra of: (**b**) Ti2p; and (**c**) O1s of BiYTi_2_O_7_.

To further determine the chemical composition of the photocatalyst BiYTi_2_O_7_, X-ray photoelectron spectroscopy (XPS) measurement was carried out. The XPS survey scan spectra of BiYTi_2_O_7_ was supplied in Figure 7. Various elemental peaks, which correspond to specific binding energies of BiYTi_2_O_7_, are provided in Table 2. The binding energies of Bi4f_5/2_, Bi4f_7/2_, Y3d_3/2_, Y3d_5/2_, Ti2p_1/2_ and Ti2p_3/2_ in BiYTi_2_O_7_ were 163.3, 156.5, 157.9, 158.6, 463.8 and 457.2 eV, respectively. The binding energies of Bi4f_7/2_ in BiYTi_2_O_7_ after carbon revision was 157.2 eV, compared with that in experimentation, revealing that the characteristic peak of Bi4f_7/2_ shifted to the lower energy. The main reason might be that the replacement of a part of Bi (2.02 for electronegativity) by Y (1.22 for electronegativity) resulted in a decrease in the electronegativity of the atoms around the Bi [75].

The consequence represented that the oxidation state of Bi, Y, Ti and O ions from BiYTi_2_O_7_ were +3, +3, +4 and −2, respectively. In addition, the average atomic ratio of Bi:Y:Ti:O for BiYTi_2_O_7_ was calculated to be the same as 1:1:2:7, based on our XPS and SEM-EDS. Consequently, it could be deduced that under our preparation condition, the resulting material was highly pure. As can be seen in Table 2, it could be found that there were two peaks with different binding energies for oxygen (O1s). The split of O1s peaks in Figure 7c was assigned to O1s peaks of crystal lattice oxygen (528.8 eV) and surface adsorbed oxygen (530.4 eV) [76]. Figure 7c shows that the XPS peak of O1s was asymmetrical, and there was acromion in the direction of higher binding energy (>530.1 eV). We fitted the O1s XPS peak, and it concluded that the Peak C1 was surface lattice oxygen species of photocatalyst, and that the Peak C2 was adsorbed oxygen on the catalyst surface after the calcination process.

#### 3.1.4. UV-Vis Diffuse Reflectance Spectra

**Figure 8:**
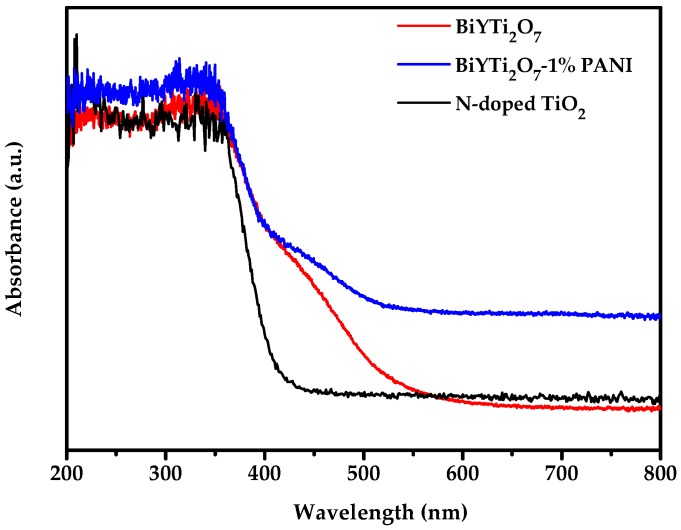
Absorption spectra of BiYTi_2_O_7_, BiYTi_2_O_7_-1% PANI and N-doped TiO_2_.

The absorption spectra of BiYTi_2_O_7_, BiYTi_2_O_7_-1%PANI composite and N-doped TiO_2_ samples are listed in Figure 8. Compared with well-known N-doped TiO_2_ whose absorption edge was no more than 420 nm, the absorption edge of this new photocatalyst was found to be at 527 nm for BiYTi_2_O_7_, i.e., at the visible region of the spectrum. Meanwhile, compared with BiYTi_2_O_7_, BiYTi_2_O_7_-1%PANI photocatalyst increased in the entire absorption spectral range.

We realized that absorbance could not be proportional to one-transmission, thus the absorbance was calculated using the Kubelka–Munk transformation method in our experiment. The optical absorption near the band edge of the crystalline semiconductors obeys the equation [77,78]:

*αhν* = A (*hν* − *E*_g_)*^n^* (1)

where A, *α*, *E*_g_ and *ν* represent proportional constant, absorption coefficient, band gap and light frequency, respectively. Within this equation, *n* determines the character of the transition in a semiconductor. *E*_g_ and *n* can be calculated by the following steps: (1) plotting ln(*αhν*) versus ln(*hν* − *E*_g_) assuming an approximate value of *E*_g_; (2) deducing the value of *n* based on the slope in this graph; and (3) refining the value of *E*_g_ by plotting (α*hν*)^1/*n*^ versus *hν* and extrapolating the plot to (α*hν*)^1/*n*^ = 0. According to the above method, the values of *E*_g_ for BiYTi_2_O_7_, BiYTi_2_O_7_-1%PANI and N-doped TiO_2_ were calculated to be 2.349, 2.476 and 2.952 eV, respectively. The estimated value of n was about 0.5 and the optical transition for these photocatalysts was allowed directly. The results showed that BiYTi_2_O_7_-1%PANI possessed a little wider band gap compared with BiYTi_2_O_7_. However, on account of the rapid charge separation from PANI with delocalized π-conjugated structures, BiYTi_2_O_7_-1%PANI could produce more electron–hole pairs and lead to higher photocatalytic activity under visible light.

#### 3.1.5. Band Structures

**Figure 9:**
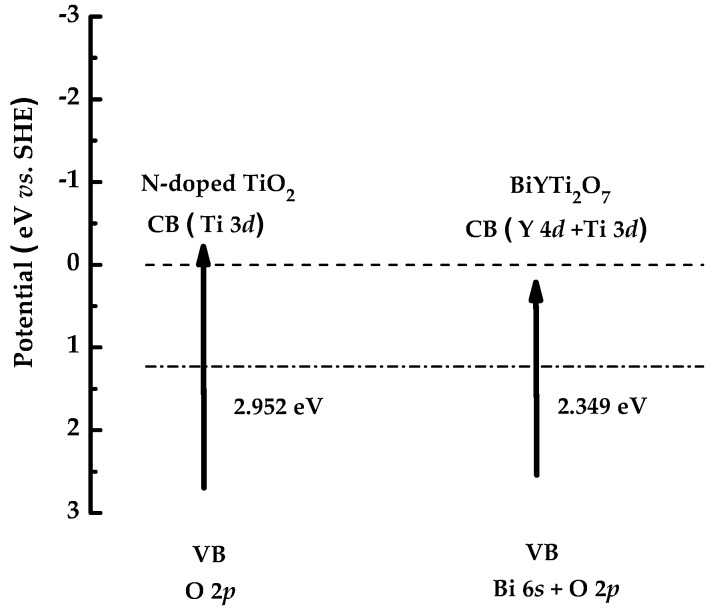
Suggested band structures of BiYTi_2_O_7_.

Figure 9 presents the advisable band structures of BiYTi_2_O_7_. Recently, Oshikiri et al. [79] reported that the electronic structures of InMO_4_ (M = V, Nb and Ta) and BiVO_4_ were described based on first principles calculations. The conduction bands of the InMO_4_ (M = V, Nb and Ta) as photocatalysts were mainly composed of a dominant d orbital component from V 3*d*, Nb 4*d* and Ta 5*d* orbitals. Meanwhile, the valence bands of the BiVO_4_ as photocatalysts were composed of a minor Bi 6*s* orbital component and a dominant O 2p orbital component. Similarly, the band structures of BiYTi_2_O_7_ should be similar to InMO_4_ (M = V, Nb and Ta) and BiVO_4_. Therefore, we deduced that, in the BiYTi_2_O_7_, the conduction band was composed of Y 4*d* and Ti 3*d* orbital component, and the valence band was composed of a small Bi 6s and O 2p orbital component. BiYTi_2_O_7_ could produce electron–hole pairs by absorption of photons directly, indicating that the enough energy which was larger than that between the conduction and valence band was necessary for decomposing AG photocatalysis process.

### 3.2. Photocatalytic Properties of Different PANI-Modified BiYTi_2_O_7_ Photocatalysts

**Figure 10:**
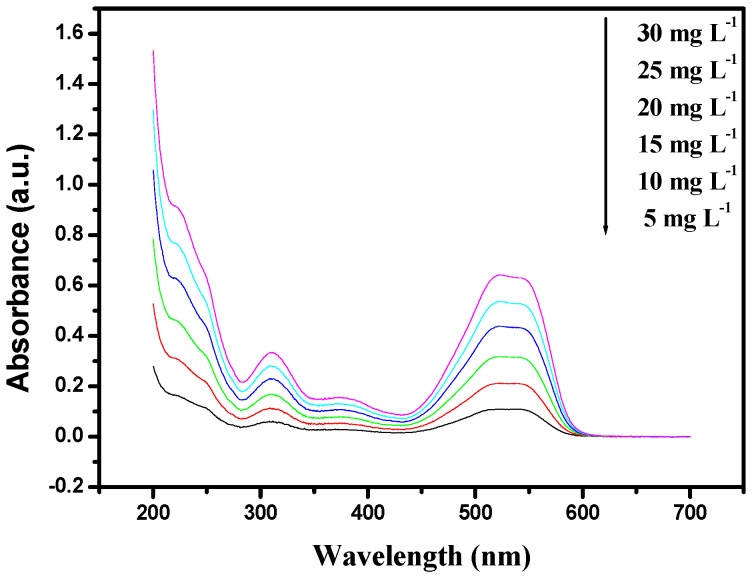
UV-Vis absorption spectra of different concentrations of AG.

**Figure 11:**
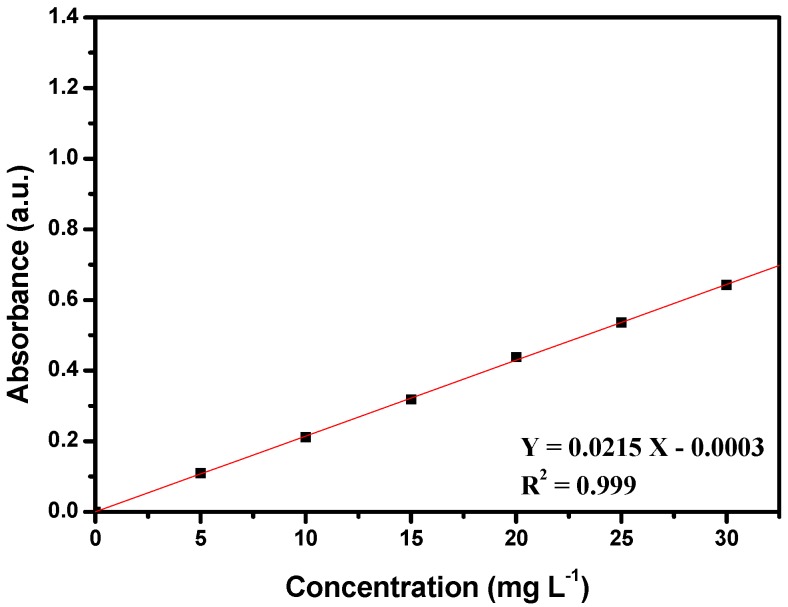
Absorbency standard curve of AG.

Figure 10 shows UV-Vis absorption spectra of different concentrations of AG. The results showed a reduction in the typical AG peaks at 524 nm. The absorbency standard curve of AG was illustrated in Figure 11. Subsequently, the standard curve equation of AG was *A* = 0.0215 *C* − 0.0003. Here, *A* and *C*, respectively, represent proportional constant and the concentrations of AG. Therefore, according to the standard curve equation of AG, the removal rate of AG was *D*% = (*C*_0_ − *C*_t_)/*C*_0_ × 100%.

**Figure 12:**
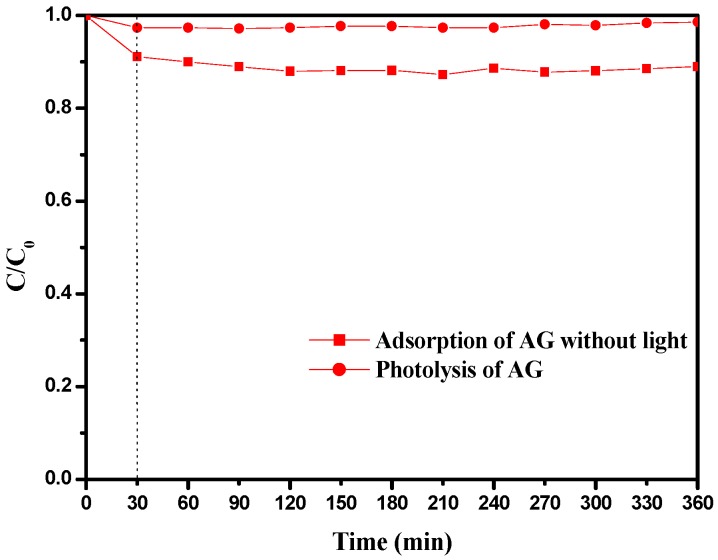
Adsorption and photolysis for AG dye.

Figure 12 shows the chart for the adsorption of AG by BiYTi_2_O_7_ in the absence of visible light and photolysis of AG without any photocatalyst. As depicted in Figure 12, the suspension had already reached adsorption/desorption equilibrium between the dye and the catalyst when stirred in the dark for 30 min. Moreover, even in the absence of a photocatalyst, the concentration of AG decreased lightly under visible light irradiation. As a result, the rate of AG degradation was estimated to be 0.22366 × 10^−9^ mol·L^−1^·s^−1^ after visible light irradiation within 360 min. It could be illustrated that the decrease on the concentration of dye without any photocatalyst was ascribed to the direct dye sensitization effect, which was similar to the observation from Liu et al. [80]. On the other side, the possible reason of slightly promoting photocatalytic effect was that BiYTi_2_O_7_ had a absorption effect for little dye without visible light irradiation. Generally, the direct absorption of band-gap photons and the generation of electron–hole pairs in the semiconductor particles leaded the photocatalytic activity [81]. Then, the charge carriers began to diffuse to the surface of the particle. Consequently, the photocatalytic activity for decomposing organic compounds with these semiconductor catalysts was enhanced.

**Figure 13:**
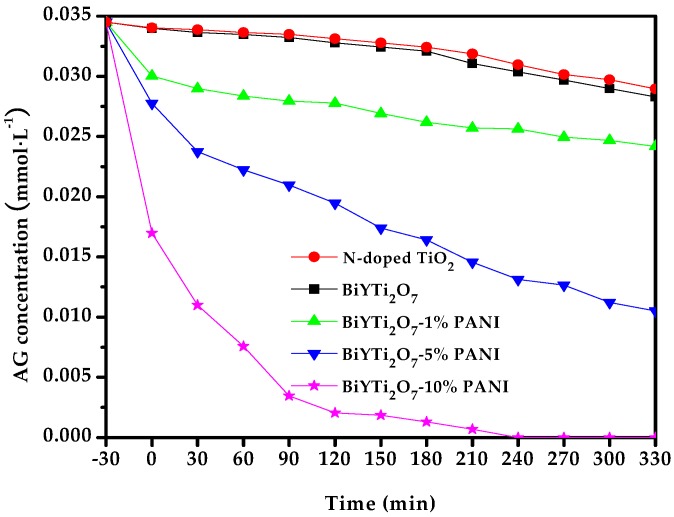
Photocatalytic degradation of AG under visible light irradiation in the presence of different PANI-modified BiYTi_2_O_7_ photocatalysts and N-doped TiO_2_.

Figure 13 is the chart for removal rate of AG (50 mL 20 mg·L^−1^) with 2 g·L^−1^ different PANI-modified BiYTi_2_O_7_ photocatalysts under visible light. The results showed that the removal rate of AG is only 18.0% or 16.1% using BiYTi_2_O_7_ or N-doped TiO_2_ as catalyst under visible light irradiation within 330 min. Similarly, the removal rate of AG was about 29.9%, 69.6% and 100%, respectively, when the BiYTi_2_O_7_-1%PANI, BiYTi_2_O_7_-5%PANI and BiYTi_2_O_7_-10%PANI were used as photocatalysts under visible light irradiation within 330 min. Additionally, the AG removal efficiency was boosted from 3.0% for undoped BiYTi_2_O_7_ to 78.0% for the 10% PANI-modified BiYTi_2_O_7_, after only 60 min of reaction. It could be concluded that the photocatalytic effects were enhanced with the improvement of polyaniline content loaded on the photocatalysts. AG was converted to small organic species and mineralized into inorganic products (CO_2_ and water) by photogenerated electrons and holes from different PANI-modified BiYTi_2_O_7_. To sum up, the photocatalytic degradation activity of these new photocatalysts was faster than common photocatalyst N-doped TiO_2_ which had a little effect on photodegrading AG under visible light. The main reason was that N-doped TiO_2_ had little response in the visible region, and the removal of AG with N-doped TiO_2_ was coming from the effects of absorption and photolysis.

**Figure 14:**
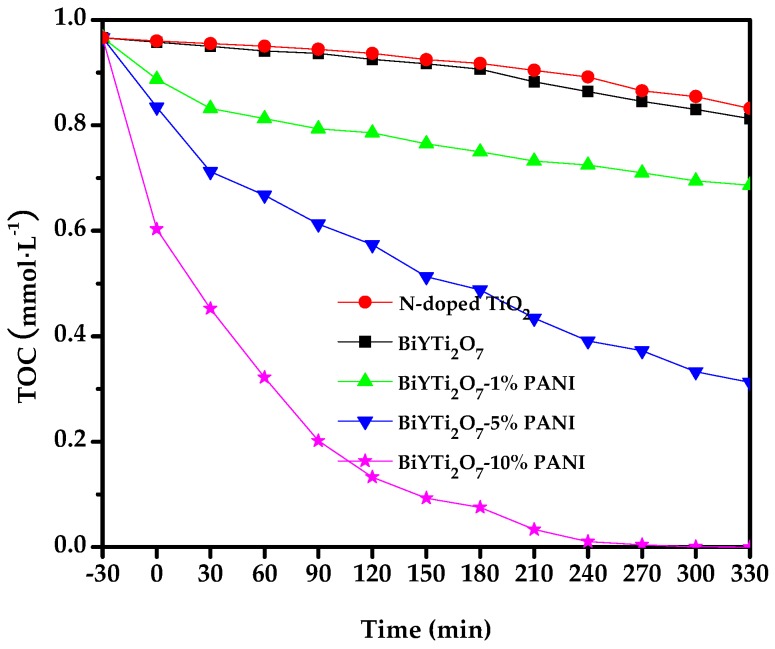
Disappearance of the total organic carbon (TOC) during the photocatalytic degradation of AG with different PANI-modified BiYTi_2_O_7_ and N-doped TiO_2_ as a catalyst under visible light irradiation.

Figure 14 presents the change of TOC for the photocatalytic degradation of AG (50 mL 20 mg·L^−1^) during visible light irradiation with 2 g·L^−1^ different PANI-modified BiYTi_2_O_7_ or N-doped TiO_2_ as a photocatalyst, which is in accordance with the tendency shown in Figure 13. The gradual decrease of TOC represented the gradual disappearance of organic carbon when the AG solution which contained different PANI-modified BiYTi_2_O_7_ or N-doped TiO_2_ was exposed under visible light irradiation. The removal rate of TOC was 15.90%, 28.93%, 65.38%, 100% and 13.87% with BiYTi_2_O_7_, BiYTi_2_O_7_-1%PANI, BiYTi_2_O_7_-5%PANI, BiYTi_2_O_7_-10%PANI and N-doped TiO_2_ as a catalyst, respectively, after visible light irradiation for 330 min. Moreover, the reactions stopped when the light was turned off in this experiment, which showed the obvious light response, suggesting that AG had been converted to other kinds of byproducts and the organic carbon in the AG had not been decomposed to CO_2_ [82].

**Figure 15:**
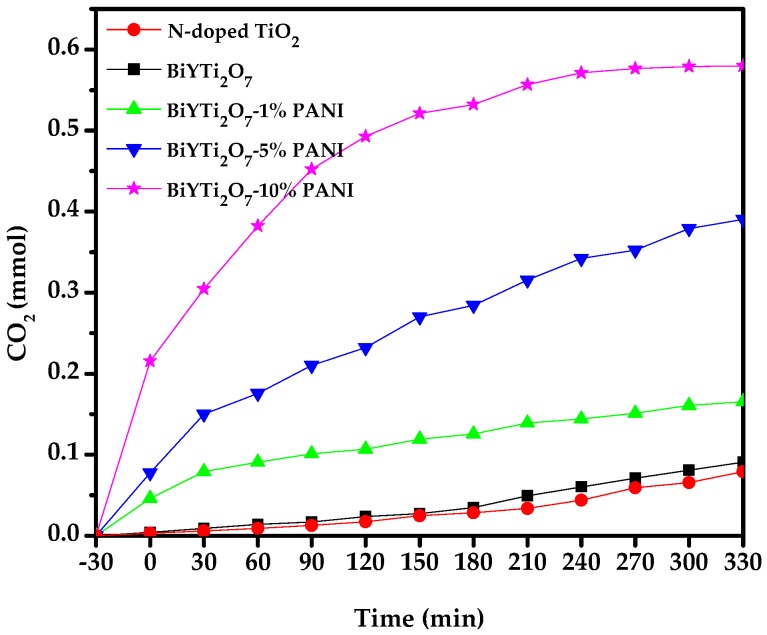
CO_2_ production kinetics during the photocatalytic degradation of AG with different PANI-modified BiYTi_2_O_7_ and N-doped TiO_2_ as a catalyst under visible light irradiation.

Figure 15 shows the amount of variation of CO_2_ produced during the photocatalytic degradation of AG (50 mL 20 mg·L^−1^) using 2 g·L^−1^ different PANI-modified BiYTi_2_O_7_ or N-doped TiO_2_ as a photocatalyst under visible light irradiation. After visible light irradiation of 330 min, the CO_2_ production of 0.09105, 0.16552, 0.39032 and 0.57958 mmol with BiYTi_2_O_7_, BiYTi_2_O_7_-1%PANI, BiYTi_2_O_7_-5%PANI and BiYTi_2_O_7_-10%PANI as a catalyst, respectively, was higher than that of 0.07925 mmol with N-doped TiO_2_. In addition, the amount of CO_2_ production was nearly equivalent to that of the removed TOC. At the same time, the amount of CO_2_ production or that of the removed TOC was slightly lower than that of reduced AG by using different photocatalysts relative to the C element equilibrium, indicating that AG was mainly degraded into some inorganic products including CO_2_ and eventually H_2_O.

There was a linear correlation between ln(*C*/*C*_o_) and the irradiation time for the (visible light) photocatalytic AG degradation with the presence of these novel photocatalysts. Here, *C* represented the AG concentration at time *t*, and *C*_o_ represented the initial AG concentration. According to Table 3, the first-order rate constant *k* of AG concentration with BiYTi_2_O_7_, BiYTi_2_O_7_-1%PANI, BiYTi_2_O_7_-5%PANI and BiYTi_2_O_7_-10%PANI were estimated. In addition, the different values of *k* indicated that PANI-modified BiYTi_2_O_7_ photocatalysts were more suitable for the photocatalytic degradation of AG under visible light irradiation than BiYTi_2_O_7_ photocatalyst.

**Figure 16:**
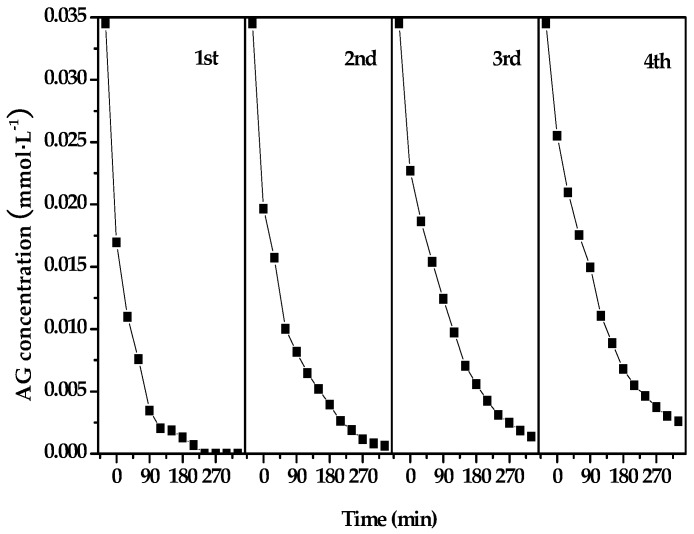
Repeated photocatalytic degradation tests of AG with 10% PANI-modified BiYTi_2_O_7_.

In order to study the stability of performance for PANI-modified BiYTi_2_O_7_ as a visible light catalyst, repeated photocatalytic degradation tests of AG with 10% PANI-modified BiYTi_2_O_7_ as photocatalyst under visible light irradiation was carried out. As can be seen in Figure 16, the removal efficiencies of AG were 98.2%, 96.0% and 92.5% for the second, third and fourth cycles, respectively, after visible light irradiation of 330 min. Figure 16 indicates that the activity slightly decreased after the first cycle, which was probably due to a small drop of 7.5% in the amount of 10% PANI modified BiYTi_2_O_7_ particles. Although the photocatalytic degradation efficiency of AG dropped from 100% for the first cycle to 92.5% for the fourth cycle, 10% PANI-modified BiYTi_2_O_7_ still showed excellent stability and was considered to be an efficient photocatalyst.

### 3.3. Photocatalytic Degradation Mechanism

**Figure 17:**
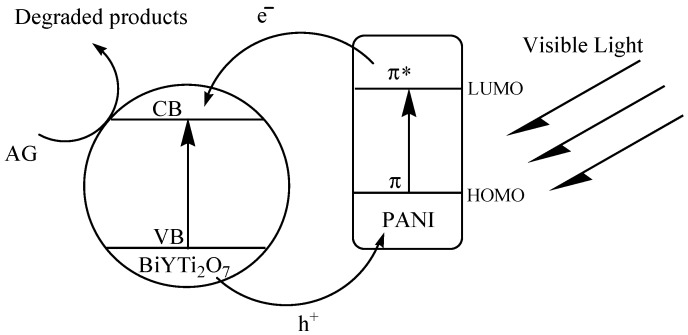
Schematic representation of the mechanism of photocatalytic degradation with PANI-modified BiYTi_2_O_7_.

Schematic representation of the mechanism of photocatalytic degradation AG with PANI-modified BiYTi_2_O_7_ is shown in Figure 17. PANI absorbed visible light to induce π–π* transition and transferred the excited-state electrons to the π*-orbital [70]. The delocalized π–π conjugated structures have been proven to induce a rapid photo-induced charge separation and to decrease the charge recombination rate in electron-transfer processes [83,84,85]. The conductive band of BiYTi_2_O_7_ and π*-orbital of PANI matched well in energy level and had chemical bond interaction [83], which could cause synergistic effect. Subsequently, based on the synergic effect, the excited-state electrons could readily inject into the conductive band of BiYTi_2_O_7_ and transfer to the surface to react with water and oxygen to yield ·OH radical and ·O_2_^−^ radical, which would oxidize the organic pollutants. In addition, the energy level of the highest occupied molecular orbital (HOMO) in PANI was between the conductive band and valence band of BiYTi_2_O_7_, therefore, these bands tended to combine to produce synergetic effect [70]. When different PANI-modified BiYTi_2_O_7_ photocatalysts absorbed visible light to generate electron–hole pairs, the holes in valence band of BiYTi_2_O_7_ could transfer to HOMO of PANI directly in virtue of a good match between the valence band of BiYTi_2_O_7_ and the HOMO of PANI. Since PANI was a good material for transporting holes, the photogenerated holes could emigrate to the photocatalysts surface easily and oxidize the adsorbed AG directly. Consequently, a rapid photogenerated charge separation and relatively slow charge recombination was achieved, which could dramatically enhance the photocatalytic activity of different PANI-modified BiYTi_2_O_7_ photocatalysts [73,86].

**Scheme 1:**
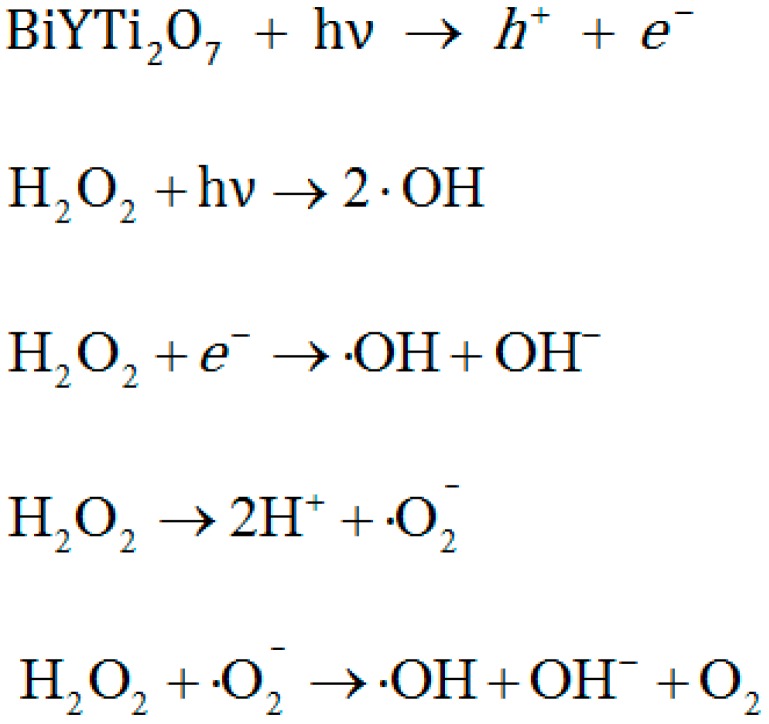
Mechanism of photocatalytic activity with H_2_O_2_ in the AG.

**Figure 18:**
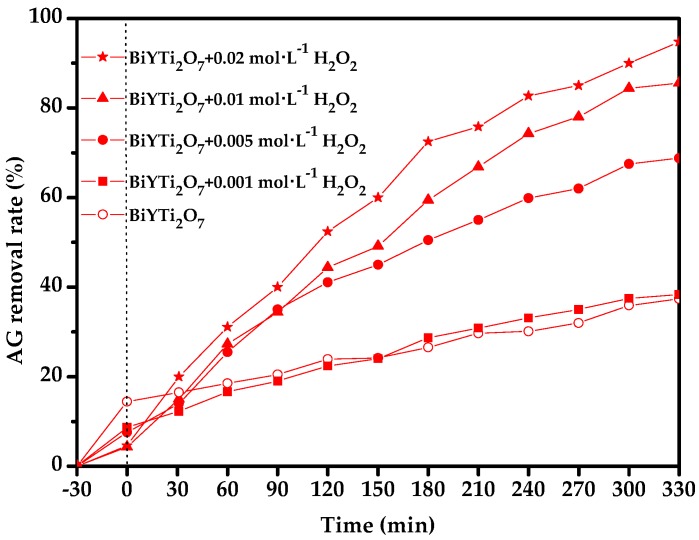
Influence of H_2_O_2_ in the degradation of AG with BiYTi_2_O_7_.

Figure 18 shows the influence of H_2_O_2_ dosage (added at −30 min) on the degradation efficiency of AG (50 mL 20 mg·L^−1^) with BiYTi_2_O_7_ (4.4 g·L^−1^). Since reactive ·OH radical was easily generated by the breakdown of H_2_O_2_, the presence of H_2_O_2_ in the reaction mixture would take great effect on the photocatalytic process in the visible light system. The increase of H_2_O_2_ concentration resulted in a faster degradation of AG. It was owing to photolysis of H_2_O_2_ to produce ·OH radical and H_2_O_2_ suitable for trapping electrons by preventing the recombination of e^−^ and h^+^ pairs [87,88]. Therefore, these phenomena increased the chance of the formation of ·OH radical and ·O_2_^−^ radical on the surface of the photocatalyst. The photodegradation rate of AG had been found to be much higher in the presence of H_2_O_2_ compared to that without H_2_O_2_. It was noted that above 90% photodegradation was achieved with 330 min in the presence of H_2_O_2_ (0.02 mol·L^−1^) instead of 37.4% degradation in the absence of H_2_O_2_. It has also been widely reported that the addition of small amount of H_2_O_2_ greatly enhances the oxidation of organic pollutants [89,90,91,92]. The decrease mechanism in the degradation of AG in the presence of H_2_O_2_ was shown in the Scheme 1.

The present results indicated that different PANI-modified BiYTi_2_O_7_ photocatalysts-visible light photocatalysis system might be regarded as a potential and practical method to treat diluted colored wastewater. This system could be used for decolorization, purification and detoxification for textile, printing and dyeing industries in the long-day countries. In addition, this sort of system did not need high pressure oxygen, heating or any other chemical reagents, which could guarantee the economic sustainability. In summary, the different PANI-modified BiYTi_2_O_7_ photocatalysts–visible light photocatalysis system might provide a valuable treatment for purifying and reusing colored aqueous effluents.

## 4. Conclusions

In the present work, we developed a solid-state reaction method for the preparation of BiYTi_2_O_7_ as a novel photocatalyst and a chemical oxidation polymerization method for the preparation of the novel polyaniline/BiYTi_2_O_7_ polymer composite at the first time. The as-prepared BiYTi_2_O_7_ crystallized with the pyrochlore-type structure and cubic crystal system (space group Fd3m). Meanwhile, by estimating, the value of the band gaps of BiYTi_2_O_7_ and BiYTi_2_O_7_-1% PANI were about 2.349 and 2.476 eV so that these new photocatalysts showed strong optical absorption in the visible light region. Photodegradation of AG aqueous solutions was observed under visible light irradiation in the presence of BiYTi_2_O_7_ and different PANI-modified BiYTi_2_O_7_ compared with normal photocatalyst N-doped TiO_2_ in this article. Additionally, the AG removal efficiency was boosted from 3.0% for undoped BiYTi_2_O_7_ to 78.0% for the 10% PANI-modified BiYTi_2_O_7_, after only 60 min of reaction, indicating that the photocatalytic effect was enhanced with the improvement of polyaniline content in the PANI-modified BiYTi_2_O_7_ photocatalysts. The AG photodegradation with these new photocatalysts followed the first-order reaction kinetics. The synergetic effect, which was caused by the good match between the conductive band of BiYTi_2_O_7_ and the delocalized π–π conjugated structures of PANI or HOMO in PANI between the conductive band and valence band of BiYTi_2_O_7_, dramatically enhanced the photocatalytic activity of different PANI-modified BiYTi_2_O_7_ photocatalysts. Moreover, the promoter action of H_2_O_2_ for photocatalystic degradation by BiYTi_2_O_7_ for AG in the wastewater was discovered. In summary, different PANI-modified BiYTi_2_O_7_–Visible system might be regarded as a potential method for treating textile industry wastewater.

## Figures and Tables

**Table 1:** Structural parameters of BiYTi_2_O_7_ prepared by solid state reaction method.

Atom	*x*	*y*	*z*	Occupation factor
Bi	0.5	0.5	0.5	0.5
Y	0.5	0.5	0.5	0.5
Ti	0	0	0	1
O(1)	0.375	0.375	0.375	1
O(2)	0.125	0.125	0.125	1

**Table 2:** Binding energies of key elements in BiYTi_2_O_7_.

BiYTi_2_O_7_	Bi4f		Y3d		Ti2p		O1s	C1s
	Bi4f_5/2_	Bi4f_7/2_	Y3d_3/2_	Y3d_5/2_	Ti2p_1/2_	Ti2p_3/2_		
Experimental (eV)	163.3	156.5	157.9	158.6	463.8	457.2	528.8	283.9
After carbon revision (eV)	164.0	157.2	158.6	159.3	464.5	457.2	529.5	284.8

**Table 3:** Kinetics parameters of photocatalytic degradation of AG with BiYTi_2_O_7_, BiYTi_2_O_7_-1%PANI, BiYTi_2_O_7_-5%PANI and BiYTi_2_O_7_-10%PANI.

Photocatalysts	*R*	Regression equation	*K* (h^−1^)
BiYTi_2_O_7_	0.9697	*Y* = 0.0337*X* − 0.0052	0.0337
BiYTi_2_O_7_-1%PANI	0.9877	*Y* = 0.0379*X* + 0.1515	0.0379
BiYTi_2_O_7_-5%PANI	0.9648	*Y* = 0.2441*X* + 0.1698	0.2441
BiYTi_2_O_7_-10%PANI	0.9874	*Y* = 0.9004*X* + 0.7508	0.9004
